# Lean Participative Process Improvement: Outcomes and Obstacles in Trauma Orthopaedics

**DOI:** 10.1371/journal.pone.0152360

**Published:** 2016-04-28

**Authors:** Steve New, Mohammed Hadi, Sharon Pickering, Eleanor Robertson, Lauren Morgan, Damian Griffin, Gary Collins, Oliver Rivero-Arias, Ken Catchpole, Peter McCulloch

**Affiliations:** 1 Saïd Business School, University of Oxford, Oxford, United Kingdom; 2 Warwick Medical School, University of Coventry and Warwick, Warwick, United Kingdom; 3 Nuffield Department of Surgical Sciences, University of Oxford, Oxford, United Kingdom; 4 Centre for Statistics in Medicine, University of Oxford, Oxford, United Kingdom; 5 Nuffield Department of Population Health, University of Oxford, Oxford, United Kingdom; 6 Cedars-Sinai Medical Centre, Los Angeles, United States of America; University of Pittsburgh, UNITED STATES

## Abstract

**Objectives:**

To examine the effectiveness of a “systems” approach using Lean methodology to improve surgical care, as part of a programme of studies investigating possible synergy between improvement approaches.

**Setting:**

A controlled before-after study using the orthopaedic trauma theatre of a UK Trust hospital as the active site and an elective orthopaedic theatre in the same Trust as control.

**Participants:**

All staff involved in surgical procedures in both theatres.

**Interventions:**

A one-day “lean” training course delivered by an experienced specialist team was followed by support and assistance in developing a 6 month improvement project. Clinical staff selected the subjects for improvement and designed the improvements.

**Outcome Measures:**

We compared technical and non-technical team performance in theatre using WHO checklist compliance evaluation, “glitch count” and Oxford NOTECHS II in a sample of directly observed operations, and patient outcome (length of stay, complications and readmissions) for all patients. We collected observational data for 3 months and clinical data for 6 months before and after the intervention period. We compared changes in measures using 2-way analysis of variance.

**Results:**

We studied 576 cases before and 465 after intervention, observing the operation in 38 and 41 cases respectively. We found no significant changes in team performance or patient outcome measures. The intervention theatre staff focused their efforts on improving first patient arrival time, which improved by 20 minutes after intervention.

**Conclusions:**

This version of “lean” system improvement did not improve measured safety processes or outcomes. The study highlighted an important tension between promoting staff ownership and providing direction, which needs to be managed in “lean” projects. Space and time for staff to conduct improvement activities are important for success.

## Introduction

Despite the extensive interest in lean approaches in healthcare [1–3] there is a shortage of evidence about the impact and execution of such initiatives [4–6]. Although attractive, the implementation of a major lean project carries with it a significant investment outlay which would pose challenges to many government funded hospitals such as those in the British NHS. There is therefore interest in whether lean can be effective when used with limited resources. The strong current focus on patient safety in healthcare has resulted in interest in using systems improvement approaches such as lean to enhance the safety of clinical work processes. The objective of this study was to evaluate the use of a light-touch intervention based around limited lean training to improve in-theatre performance and clinical outcome in orthopaedic trauma surgery.

This intervention was performed as part of a multi-site research programme (Safer Surgical Services—S3), whose aims were to compare the effectiveness of safety and quality interventions in surgery based on improving safety culture with those based on improving systems of work, and to determine whether using both types together had an additive or synergistic effect. In each of five identically designed controlled studies we used the same observational process methods to evaluate theatre team’s non-technical skills (Oxford NOTECHS II)[7]; intra-operative process disruptions (glitch)[8], and WHO surgical safety checklist compliance[9], and hospital statistics to evaluate clinical outcomes. Each study tested the effect of a different intervention or combination of interventions.

One substantial challenge in conducting rigorous research in this field is the difficulty of defining Lean systematically[10, 11]. Various writers emphasise different aspects, and the definitions used in practice may be fluid and vague. We have here adopted a view influenced by Spear and Bowman[12] which emphasises experimentally-oriented, participative problem solving and process improvement, in contrast to the top-down implementation of specified best practices[13]. Our previous research [14] highlighted important strengths of bottom-up, participative approaches to process improvement, with ownership of the processes of problem analysis and solution implementation by the workforce, and we therefore continued with this approach in the present study[14]. This approach emphasises the idea of people applying creativity to their working practices (‘challenge’), working towards a constant cycle of improvement in (sometimes small) experimental steps (‘Kaizen’) driven by detailed analysis of the working practices (‘Genchi Genbutsu’), in the context of respectful cooperation between team members, regardless of status or seniority (See Appendix). This approach to lean was developed by our multidisciplinary team as part of the S3 programme, and was implemented by staff coached by team members in both S3 studies where lean was used[REF other one].

## Methods

### Setting and overview

This study took place in the Trauma and Orthopaedic services division of a large UK teaching hospital. The department consists of over twenty consultants working across three sites. We selected for intervention the Trauma services theatre team for the same day each week, as this comprised a more or less stable group of staff based on a small number of identifiable consultant surgeons. The control group was a set of consultant teams performing elective orthopaedic surgery at a different hospital site within the same Trust, which was physically some miles distant and was dedicated to elective orthopaedic surgery and other routine planned treatments During the period of the study there was no cross-membership to contaminate the experiment. The patients and conditions operated on in the two teams were typical of the kind of work performed in such theatres in the NHS—principally consisting of fixation of common fractures in the trauma theatre and replacement of arthritic joints in the routine orthopaedic theatre.

### Study design

The study was designed as a controlled interrupted time series, with 6 months pre intervention data collection, 6 months intervention (active only) and 6 months post intervention data collection. This study was one of 5 identically designed intervention experiments which formed a research programme investigating the strengths and weaknesses of two categories of intervention to improve patient safety by making clinical team processes more reliable. The larger programme included experiments evaluating interventions addressing team culture (using aviation-style crew resource management training), a different approach to systems improvement, and combinations of a system and a culture intervention.

### Ethics

Patients whose operations were observed were informed of the possibility of observations taking place and given opportunity to opt out if they wished. Staff in the theatres undergoing observation were given information on the study and asked for consent before observations took place. The study was approved by Oxford A Ethics Committee (REC:09/H0604/39).

### Primary and secondary interventions

The intervention we performed can be viewed on two levels. We describe the methodologies and results separately here to avoid confusion. The *primary* intervention consisted of training in lean theory and methods, and subsequent expert support and encouragement. For this intervention, we adopted a rigorous methodology involving systematic data collection of pre-defined metrics, enabling comparison with a control group and with other interventions within the S3 study programme. The focus of the overall programme was improvement of team processes relevant to safety, and three relevant process measures were therefore pre-defined and used in all S3 studies, regardless of whether they reflected the direct focus of work chosen by the clinical teams.

The *secondary* intervention consisted of the improvement exercise that the primary intervention then stimulated. This comprised observational study of patient and information flow, analysis, and implementation of a series of changes to practices. The secondary intervention was principally the work of the surgical team, with some support from the research team. It adopted the pattern of data collection and analysis common in participative process improvement, including analysis of very small datasets to monitor and modify process (PDCA cycles).

### Primary intervention

Staff attended a one day Lean training course, followed up by light-touch coaching for 6 months, combined with some assistance in data collection and analysis. The course was delivered to twenty team members, including surgeons, nurses, administrators and anaesthetists. It covered the principles of Lean, including Muda, Poka-Yoke, Genchi Genbutsu, Kaizen, flow, Just in time, respect and teamwork, process mapping, PDCA cycles and a philosophy of continuous participative experimental improvement. (See Appendix). It was delivered by a lean specialist with experience of working in hospitals (SN). The session concluded with the team identifying an area for process improvement and developing an action plan. A small group of four staff were unable to attend on the main day, and an extra half-day session was therefore run a few days earlier for them. Preparation for the training day included a pre-course visit and consultation with the observers who collected pre-intervention performance data (described below). Although staff attended only one formal day of classroom teaching about lean, those involved in the secondary intervention subsequently received further practical training and instruction in its use during their activities on a weekly basis. The “dose” of training and coaching in lean techniques was therefore considerably more than one person-day per staff member, but was hard to quantify.

### Process evaluation: standard measures

A large convenience sample of operations (those occurring on a given weekday) in both Intervention and Control groups was observed from beginning to end, to evaluate team technical and non-technical performance and compliance with WHO checklist procedures. These observations were confined to the 3 months immediately before and immediately after the intervention period. Each operation was observed by two observers; one with a clinical (surgical) and one with a human factors (HF) background. The clinical observers included two surgical trainees (MH, ER) and one nurse practitioner (JM). The HF specialists all had a higher degree in human factors and / or psychology (SP, LM, LB). Prior to the commencement of the study a two month training phase was completed by all observers to aid familiarisation with the data collection methodologies. Intra-operative observation began when the patient entered theatre and ended when they left it. Data collection booklets for each surgical procedure were developed to record observational data. We assessed the effects of the intervention on work processes relevant to safety with three observational outcome measures. Team non-technical skills were assessed using the Oxford NOTECHS II method[7], technical operative process reliability was assessed using the “glitch count” method[8] and functional compliance with the WHO checklist process was evaluated using a simple observational method[9]. These methods were developed specifically for the S3 programme and were tested for reliability and validity prior to use.

#### Oxford NOTECHS II

The operating team’s non-technical skills were assessed using the Oxford NOTECHS II behavioural rating scale[7]. Each sub-team; nursing, surgical and anaesthetic were scored on a 1–8 scale against four behavioural parameters: leadership and management; teamwork and cooperation; problem solving and decision making; and, situational awareness[7, 15]. Mean whole-team Oxford NOTECHS II score was estimated for all operations observed before the intervention and compared to the same score for operations observed after it, in both intervention and control groups.

#### Glitches

Glitches are defined as “deviations from the recognised process with the potential to reduce quality or speed, including interruptions, omissions and changes, whether or not these actually affected the outcome of the procedure”[8]. These were noted independently by each observer, noting the time and details of each glitch (e.g. ‘diathermy not plugged in when surgeon trying to use it’). Following the completion of the operation, the glitches were categorised and entered into a secure database. A final glitch score was subsequently decided on by agreement and a glitch rate per hour of operating time was calculated for each operation. The mean glitch score for operations observed before and after the intervention were compared for both intervention and control groups.

#### WHO Surgical Safety Checklist

We observed the time-out (T/O) and sign-out (S/O) sections of the WHO Surgical Safety Checklist for each operation studied. We recorded whether these were attempted, and where they were, recorded three measures of quality: (1) whether all information was communicated, (2) whether all team members were present and (3) whether there was active participation by team members[9]. Observers agreed and entered a final combined score as previously described[2].

#### Clinical Outcome evaluation

Anonymised clinical outcome data on readmissions within 90 days, complications and length of stay were extracted from hospital records. We obtained ethics clearance to extract non-identifiable individual patient-level data from all patients under the care of the consultants participating in the S3 study. For each consultant in the active or control group, clinical outcome data were obtained for all his/her patients for six months before and six months after the intervention was delivered. In order to ensure anonymity and to avoid linking consultants to a particular case, the consultant data were combined into intervention and control groups.

### Secondary intervention

In discussions at the training day, the topic that emerged as of greatest concern to staff was delay to the start of the operating list. There was consensus that this not only impacted efficiency but also potentially patient safety: delays at the start of the day can lead to later operations being postponed and to further patient scheduling changes, causing confusion and tension that was perceived to increase the probability of other kinds of errors. It was therefore decided to focus improvement efforts on an attempt to improve theatre start times, rationalise the order of cases and minimise delays. The methods of evaluation in this secondary intervention flowed from the decisions about improvements to make and are described in the relevant part of the Results section.

The group discussion led to the identification of tasks required to allow exploration of process improvements. These were allocated to individuals but for reasons discussed below, momentum rapidly dissipated. A sub-group of team members led by a consultant surgeon then moved to revitalise the initiative, and devised a data collection exercise to establish a more detailed understanding of the problem.

The key issue that became clear was the multiplicity of causes of delay, and the difficulty of identifying which merited action. Causes included inadequate preparation of patients for movement to theatre; the order in which anaesthetists undertook pre-operative tasks; and delays and failures in communication between the pre-list meeting (the ‘trauma meeting’), the operating theatre, and the ward. Staff shared a sense of frustration and a tendency for different professional groups to blame others for the difficulties. A scheme was therefore designed with observers at key locations (operating theatre, the pre-list meeting room, and on the relevant wards) recording the sequence of events from the beginning of the day to the beginning of the second operation. Forms were designed which enabled the systematic collection of timing data. The data items collected included timings for the post-take word round process and for the patient journey from ward to theatre through to the start of the first operation of the day, effectiveness of liaison with anaesthetists and changes to the pre-planned list order.

**Fig 1:**
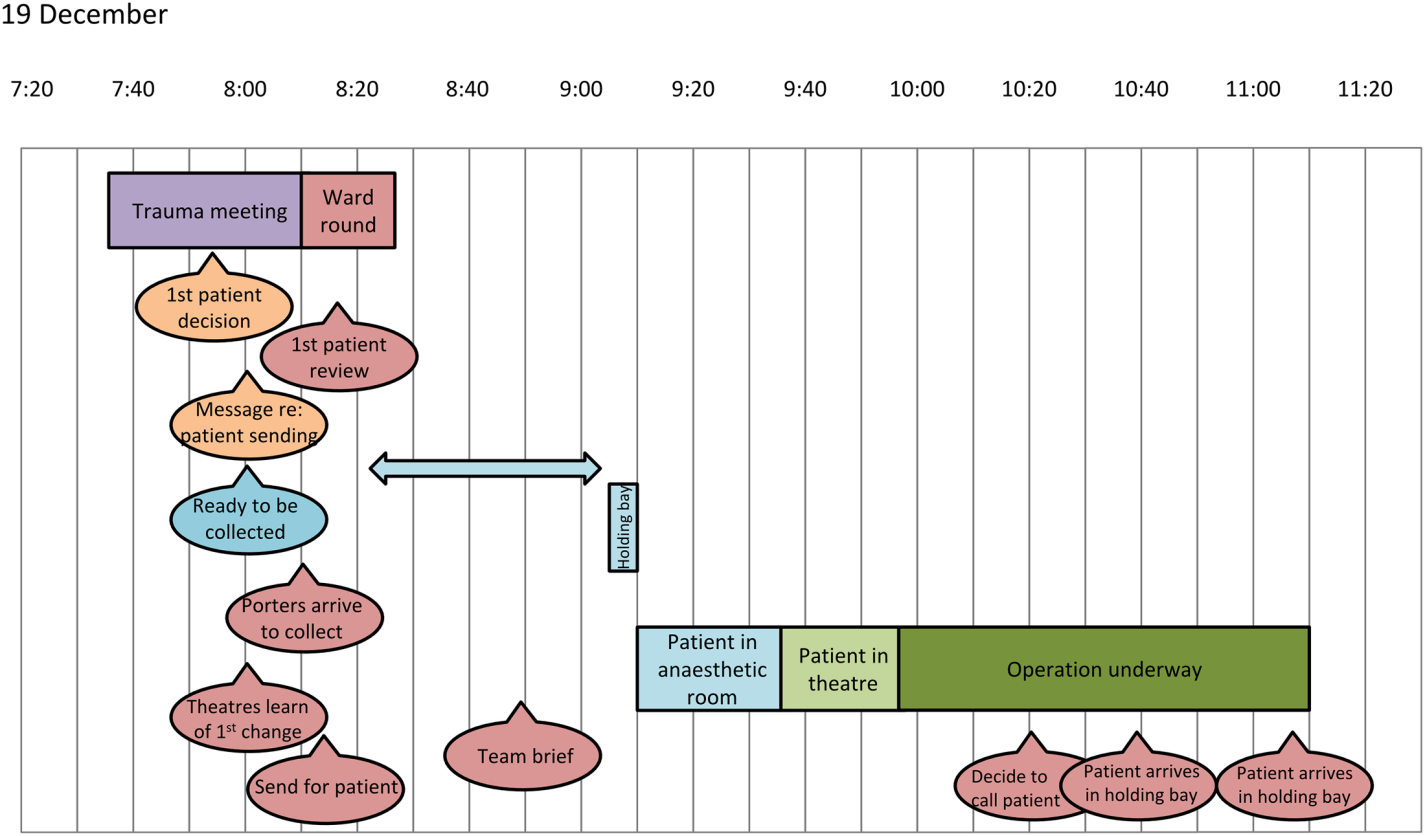
Example Patient Flow Chart.

The consultant galvanised a group of helpers—trainee doctors and specialist nurses, augmented by members of the research team—to capture data over a two-week period and generate time charts (Fig 1), to establish how long things took and to gain a deeper understanding of the process. This data informed meetings with subgroups, and a lively exchange of emails amongst an extended group of staff. This, in turn, led to an experimental change to a standard working pattern, including new patterns of communication after the pre-list meeting, new protocols for organising the meeting, and guidelines for sequencing and timing activities.

The observational data collection process was then repeated in March 2012 allowing a comparison of the timings data before and after intervention.

#### Data analysis

Differences in before-after intervention change between the control and active arms were assessed using two-way analysis of variance (group × time), with treatment (control versus active) and time (pre-intervention versus post-intervention) as factors. Differences between groups were assessed by the group x time interaction. Pre- and post-intervention differences are reported as 95% confidence intervals. All statistical analyses were carried out in R (version 3.0.1). For clinical outcome data, baseline demographic information was summarised using descriptive statistics. T-tests for mean age and chi-square test for gender distribution were used to compare the before and after periods. Binary clinical outcome variables in the before and after periods were compared using Odds ratios and 95% confidence intervals from a logistic regression adjusted for age and gender. These variables included “dead within 30 days”, “readmission within 90 days” and having at least one complication. Mean length of stay in the before and after periods was compared using linear regression controlling for age and gender. In both regression strategies the coefficient of interest was associated with a dummy identifying the before or after period and separate regressions were conducted for active and control groups. Given the number of before and after comparisons performed a 1% significance level was selected. This statistical analysis was conducted in Stata version 12.

## Results

### Primary intervention

We observed 17 operations in the active group before the intervention and 21 in the control group, compared with 25 and 16 respectively afterwards. We reviewed the records of 224 patients operated on in the active theatre and 352 in the control theatre before the intervention, and 292 and 173 patients respectively afterwards. The mean operating time was slightly shorter in the control group (1hour 45minutes compared with 2hours) but did not change by more than 5minutes in either group following the intervention.

#### Oxford NOTECHS II

**Fig 2:**
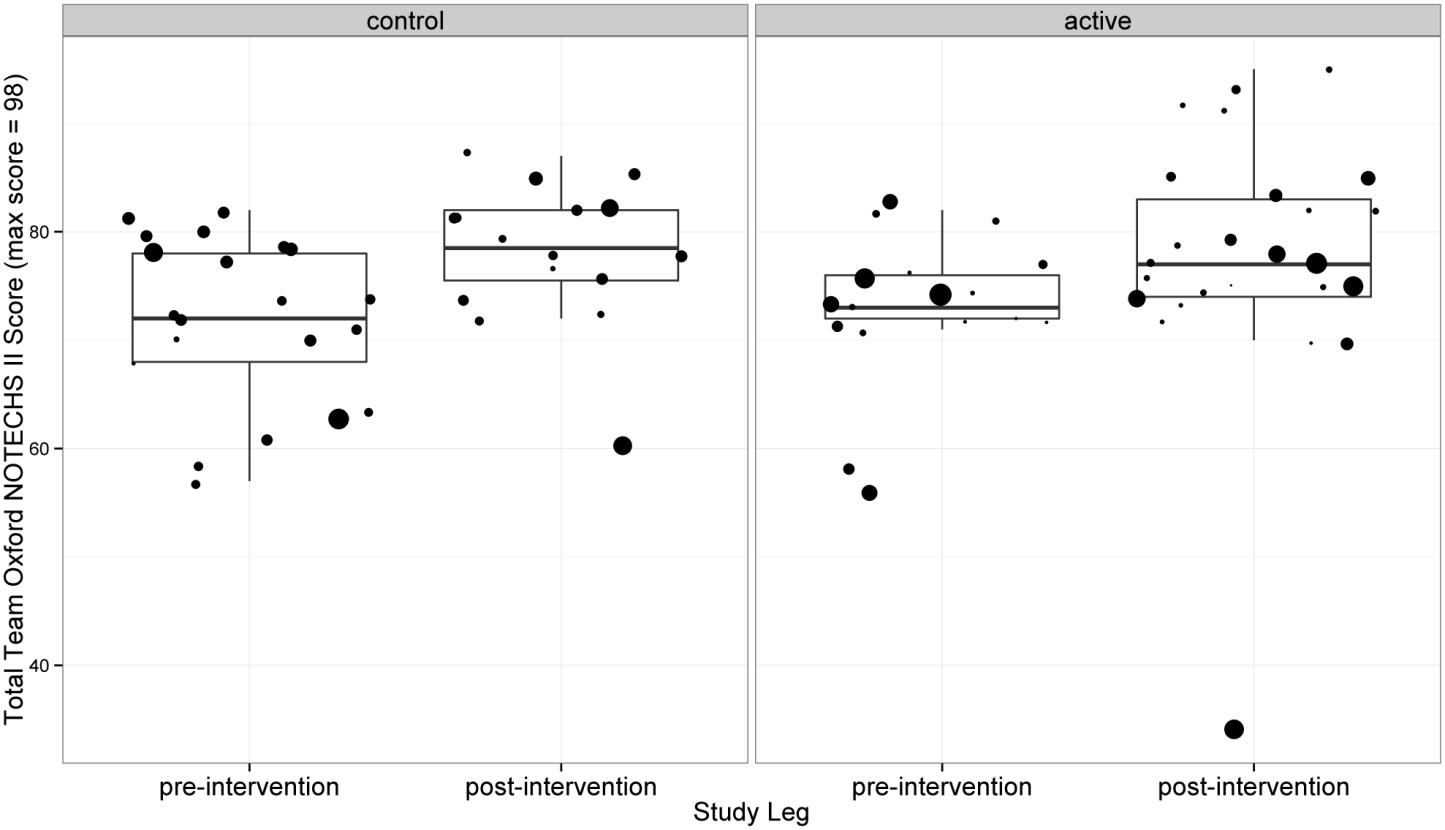
NOTECHS II Results. Each dot is the individual Total Oxford NOTECHS II score for an individual operation, and the size of the dot represents the duration of the operation.

The mean NOTECHS score increased from 73 before to 77.84 after the intervention in the active group, but also increased (from 71.31 before to 78.06 after) in the control group (Fig 2). The difference between the change in the active and control groups was not statistically significant (p = 0.938; difference 0.22; 95% CI -5.53 to 5.97). Sub team analysis showed no significant changes in mean NOTECHS scores for surgeons (p = 0.462), nurses (p = 0.803) and anaesthetists (p = 0.483).

### WHO compliance

In 79 observed operations, teams attempted T/O in 77, but attempted S/O in only 2. These figures precluded any possibility of finding inter-group or pre-post differences. Consequently the difference between the change in the active and control groups was not significant (difference -2%; 95% CI -18% to 14%; p = 1). The quality of WHO checklist completion, as assessed by our measures, is shown in Table 1.

**Table 1:** Completion of WHO Checklist.

		Pre-intervention	Post-intervention
**Control**	**Time-out performed**	20/21 (95%)	16/16 (100%)
	*Communication*	16/21 (76%)	12/16 (75%)
	*All team present*	15/21 (71%)	15/16 (94%)
	*Active participation*	18/21 (86%)	15/16 (94%)
	**Sign-out performed**	0/21 (0%)	1/16 (6%)
**Active**	**Time-out performed**	17/17 (100%)	24/25 (91%)
	*Communication*	7/17 (41%)	13/25 (52%)
	*All team present*	9/17 (53%)	17/25 (68%)
	*Active participation*	11/17 (65%)	20/25 (80%)
	**Sign-out performed**	0/17 (0%)	1/25 (4%)

There was an increase in all three measures in the active group but also in two of the three in the control group. All three components of T/O were completed in 3/17 (18%) cases in the pre-intervention active arm, which increased to 9/25 (36%) in the post-intervention phase (difference = 18%; 95% CI -13% to 49%). All three components of T/O were completed in 11/21 (52%) cases in the pre-intervention control arm, which increased to 10/16 (62%) in the post-intervention phase (difference = 10%; 95% CI -27% to 48%). The difference between the change in the active and control groups was not significant (p = 0.621).

### Glitch counting

**Fig 3:**
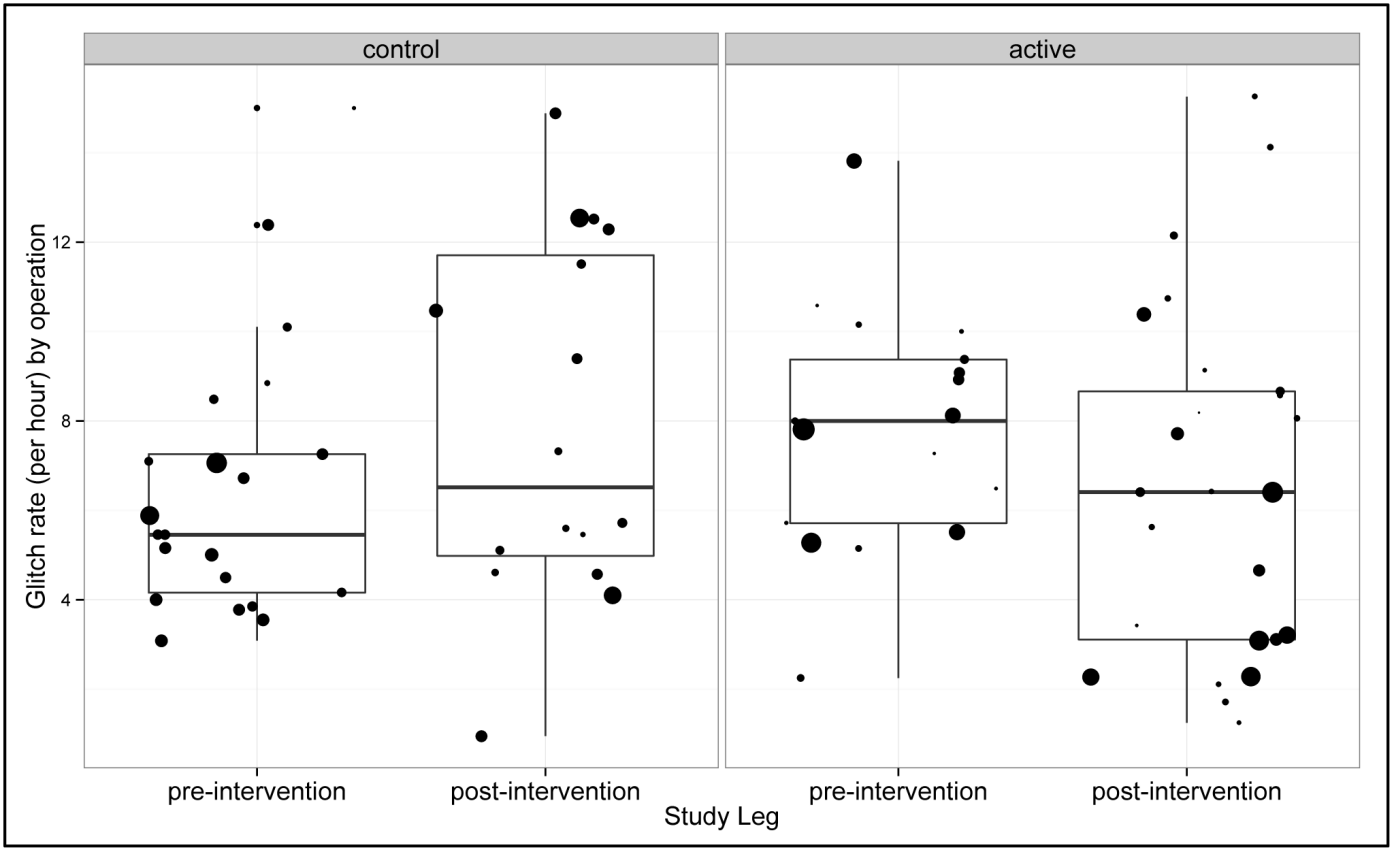
Glitch Rates. Each dot is the individual Glitch Rate for an individual operation and the size of the dot represents the duration of the operation.

The mean glitch rate per operation was 7.85 (sd = 2.69) glitches per hour before the intervention in the active group, and decreased to 6.59 (sd = 3.95) glitches per hour afterwards (difference = -1.26; 95% CI -3.33 to 0.81). The rate in the Control group began lower at 6.52 (sd = 3.06) glitches per hour, but increased after the intervention to 7.94 (sd = 4.01) glitches per hour (difference = 1.42; 95% CI -1.05 to 3.90). The difference between the change in the active and control groups was not statistically significant (p = 0.098) (Fig 3).

### Clinical outcomes

In the six months prior to the intervention, 224 patients were operated on in the Intervention theatre and 352 in the control theatre, compared with 292 and 173 respectively afterwards. The reversal in activity rates between the two theatres is explained by economic changes which resulted in a Trust decision to cut back on elective orthopaedic surgery. The complication rate in the intervention group increased very slightly and that in the control group decreased somewhat more (Table 2) but the difference in before/after ratios is not significant. The length of hospital stay declined from around 10 days to around 7 in both groups. This may have been related to a Trust initiative to cut length of stay across multiple specialities, which was conducted independently. In neither group was there any noticeable change in readmissions to hospital.

**Table 2:** Main process and outcome results.

	Active		Control		P value (Δ control VS Δ INTERVENTION)
	Pre-intervention	Post-intervention	Pre-intervention	Post-intervention	
NOTECHS Mean (SD)	73 (7.1)	77.84 (11.59)	71.81 (7.72)	78.06 (6.57)	0.938
WHO Time Out attempt	17/17 (100%)	24/25 (96%)	20/21 (95%)	16/16 (100%)	1
WHO Time-Out complete compliance	3/17 (18%)	9/25 (36%)	11/21 (52%)	10/16 (62%)	0.621
WHO Sign Out	0/17 (0%)	1/25 (4%)	0/21 (0%)	1/16 (6%)	1
Glitch rate/hour	7.85 (2.69)	6.59 (3.95)	6.52 (3.06)	7.94 (4.01)	0.098
90-day Readmissions	94 (20%)	102 (18%)	130 (19%)	55 (18%)	0.3
Complications	47 (10%)	70 (12%)	95 (14%)	32 (10%)	0.07
Length of Stay in days (SD)	10.3 (25)	7.7 (15)	10.2 (20)	7.6 (16)	0.396

#### Summary of primary outcome measure results

These are shown in Table 2. As noted above, the only striking difference between the active and control groups was in glitch count, which declined in the active but increased in the control group, and this was not statistically significant.

### Secondary intervention

A repeat data collection exercise in March 2012 identified a number of apparent improvements, although with only 10 and 9 cases evaluated before and after the intervention, these were not amenable to formal statistical analysis. The improvements implemented lean principles by; reducing waste; introducing measurement; standardising work processes and showing respect for the workforce. A large number of the measures showed some improvement. For example, the presence of anaesthetists at the trauma meeting rose from 33% to 66%; the number of changes to the list was substantially reduced; and patients were reaching the anaesthetic room twenty minutes earlier. The changes did not, however, lead to improvement in the average time for ‘knife to skin’ for the first patient, due to an increase in the time spent in the anaesthetic room. A summary of improvements is shown in Table 3.

**Table 3:** Summary of changes following secondary intervention

	Dec-11	Mar -12
Mean post take ward round (PTWR) start time	08:12	07:59
Mean PTWR first patient review time	08:16	08:05
Mean PTWR second patient review time	08:41	08:08
Any communication with anaesthetist? (Y/N)	30%	44%
% of days on which operating plan was changed after PTWR	20%	0%
Patient reviewed by anaesthetist/ other?	40%	100%
Did first patient on printed list remain first on list? (Y/N)	50%	100%
Did the plan change from the printed plan during the trauma meeting?	70%	22%
Mean time porters/staff arrive to collect first patient from ward	08:18	08:10
Mean time first patient left ward	08:29	08:15
Mean time patient arrived to anaesthetic room	08:48	08:25
Mean time patient entered theatres	09:11	09:22
Mean time surgeon in theatre for case	08:46	08:38
Mean time prep started	09:22	09:29
Mean time knife to skin	09:37	09:37

## Discussion

Lean initiatives in health care have been controversial in terms of the tension between ‘top-down’, management-led initiatives and approaches which seek to exploit the knowledge and enthusiasm of staff[16, 17]. Indeed, some projects have faced explicit resistance from medical staff [18]. Health applications of lean have generally sought improvements in cost and efficiency, but some have sought to examine the use of lean to improve patient safety[19]. Literature reviews[20, 21] show that “lean” initiatives in hospitals have rarely been system-wide or long term: whilst we agree with these authors that this would be desirable, there is still very little adequately controlled data about the effects of smaller scale lean interventions. Our study showed no statistical improvement in our chosen measures of process and outcome versus a contemporary control group, and so our hypothesis that a relatively light touch “lean” training intervention would improve safety was not supported. Although the secondary intervention generated an appreciable sense of progress among staff, the results were insufficient to convincingly demonstrate functional process improvement. However, in both cases, the outcomes raise important issues.

There are several possible explanations of the findings of the pre-defined measures for the primary intervention. We relied on the surgical team identifying potential process improvements and having the capacity and motivation to engage in improvement without additional incentives. Although supported by the hospital administrators, our intervention did not form part of an official programme led by management. It was notable that several participants openly expressed cynicism about process improvement, having previously experienced ineffective and patronising initiatives. Second, our lean intervention, offering only a single day of classroom training, may have represented an insufficient dose, especially in the light of previous (negative) staff experience of process improvement initiatives. In addition, five months may have been too short a period for the cycle of innovation and experimentation to bear fruit. Many similar training initiatives are more intensive and longer, and more comprehensive. On the other hand, we did provide ongoing support, and in previous studies, we have experience of this type of abbreviated intervention being effective[14]. We also wished to avoid trialling an intervention too intensive, and therefore costly, to be a viable option for wide dissemination. We were unable, in our review of the literature to identify either convincing evidence or a clear consensus on the “dose” of lean training and coaching support required to ensure success and we therefore based our intervention on the maximum we could reliably deliver with our resources and particularly with the amount of time off the clinical staff were permitted for training and improvement activity.

The controlled design was a strength of this study, protecting against the possibility of interpreting secular trends as effects of improvement activity. The fact that the experimental and control groups were not perfectly matched was not a major drawback, since we were interested in the degree of before/after change rather than direct comparison of measures. Both groups were however subject to changes in the hospital over which the study team had no control, introducing a degree of “noise” in the results which might have obscured small intervention effects.

Another interpretation is that this version of Lean is invalid or inappropriate for the setting and type of problem. This proposition needs to be considered alongside the extensive evidence of benefits from Lean in general, and the apparent success that training of the sort deployed here has had in other settings, including in our hands[22, 23].

Another plausible explanation is that the specific focus of improvement activity was simply not causally connected with safety as measured by our chosen outcome metrics. Although a rationalised process might lead indirectly to better non-technical teamwork performance and lower glitches in the theatre, this connection might be simply be too weak to observe in a study of this scale. This seems plausible given the decision of the clinical team to focus on an objective which appeared to us more efficiency than safety-oriented. This in turn was a consequence of our decision to allow staff to set the agenda, perhaps without giving them adequate boundaries. In hindsight it might have been possible to steer the members of the team away from start time as the focus for their efforts, and towards a goal which would more directly impact on patient outcomes. However, allowing participants to identify their own problems and devise their own solutions is crucial to the logic of this version of Lean, and the strong staff engagement it can build has contributed importantly to the sustainability of solutions in previous interventions. To impose our own agenda would have risked losing ownership of the work by the people involved. Further work is required to determine how to resolve this tension between maintaining control of direction and losing staff ownership and engagement in Lean healthcare interventions.

Two significant barriers to improvement activity were the fragmented and fluid nature of the team and the lack of a convenient physical space for process improvement activity. Effective *collective* action after training proved difficult because making change required the involvement of a disparate group of staff in various parts of the Trust, some of whom never routinely met each other. Some key staff were absent at the main training day where the plan was agreed, having attended the preceding alternative session. In the subsequent days, follow-up did not happen as intended: there was slippage in the agreed timescales, miscommunication and loss of momentum. The agreed plan of action effectively evaporated, and two weeks later, progress had effectively halted. Only the initiative of a single consultant revived the momentum.

Regarding space, there was neither a room nor time in the weekly schedule for collective deliberation. From other studies in this Programme we recognise this as a common feature of modern surgical work, and one which, if solved, would greatly facilitate improvement work. We believe this to be an important learning point from this study. In this study, the fact that any progress was made at all hinged on the emergence of a particularly enthusiastic and authoritative individual in the group who was able to marshal resources to move the project along.

The data collection initiative and associated experimental approach to process improvements reflected several features of the ‘lean’ approach. It was data-driven, participative and experimental. It achieved some measure of improvement, but did not ‘solve’ the identified problem, and although the first patient was arriving earlier at the theatre, the start of the actual operation remained persistently late because of an increase in time spent in the anaesthetic room. The cause for this needs to be investigated—a comment which perhaps illustrates the unfinished nature of the project, as well as the complexity of the operational and behavioural problems which face surgical operations.

## Conclusions and Recommendations

This Lean-style process improvement failed to generate significant improvements in theatre team performance or patient outcome, but succeeded in stimulating specific process improvements. Future research should address the problem we identified of balancing control of direction with staff ownership and engagement in participative Lean processes and clarify the minimum time and space conditions needed for coherent team co-operative improvement activity to develop in clinical environments.

## References

[1] Black J.R. and Miller D.J., *The Toyota way to healthcare excellence*: *increase efficiency and improve quality with Lean*. 2008, Chicago: Health Administration Press.

[2] Aherne J. and Whelton J., *Applying Lean in Healthcare*: *A Collection of International Case Studies*. 2010: Taylor & Francis.

[3] Bercaw R.G., *Lean Leadership for Healthcare*: *Approaches to Lean Transformation*. 2013: Taylor & Francis.

[4] Blumenthal D. and Kilo C.M., A report card on continuous quality improvement. Milbank quarterly, 1998 76(4): p. 625–648. 9879305 10.1111/1468-0009.00108 PMC2751093

[5] Øvretveit J. and Gustafson D., Evaluation of quality improvement programmes. Quality and safety in health care, 2002 11(3): p. 270–275. 12486994 10.1136/qhc.11.3.270 PMC1743631

[6] Joosten T., Bongers I., and Janssen R., Application of lean thinking to health care: issues and observations. International Journal for Quality in Health Care, 2009 21(5): p. 341–347. 10.1093/intqhc/mzp036 19696048 PMC2742394

[7] Robertson ER, Hadi M, Morgan LJ, Pickering SP, Collins G, New S et al, Oxford NOTECHS II: A Modified Theatre Team Non-Technical Skills Scoring System. PLoS One, 2014 9(3): p. e90320 10.1371/journal.pone.0090320 24594911 PMC3942429

[8] Morgan L, Robertson E Griffin D Catchpole K, Pickering S, New S et al, Capturing intraoperative process deviations using a direct observational approach: the glitch method. BMJ open, 2013 3(11): p. e003519 10.1136/bmjopen-2013-003519 24282244 PMC3845041

[9] Pickering S, Robertson E, Griffin D Hadi M, Morgan LJ, Catchpole K et al, Compliance and use of the World Health Organization checklist in UK operating theatres. British Journal of Surgery, 2013 100(12): p. 1664–1670. 10.1002/bjs.9305 24264792

[10] Liker J.K., *The toyota way*. 2004: Esensi.

[11] Graban M., *Lean hospitals*: *improving quality*, *patient safety*, *and employee satisfaction*. 2011: CRC Press.

[12] Spear S. and Bowen HK, Decoding the DNA of the Toyota production system. Harvard Business Review, 1999 77: p. 96–108.

[13] Liker J. and Franz JK, *The Toyota Way To Continuous Improvement*: *Linking Strategy And Operational Excellence To Achieve Superior Performance Aut*. 2011.

[14] McCulloch P, Kreckler S, New S Sheena Y, Handa A, Catchpole K. QUALITY IMPROVEMENT REPORT: Effect of a" Lean" intervention to improve safety processes and outcomes on a surgical emergency unit. BMJ: British Medical Journal, 2010 341(7781). 10.1136/bmj.c5469 21045024

[15] Mishra A., Catchpole K., and McCulloch P., The Oxford NOTECHS System: reliability and validity of a tool for measuring teamwork behaviour in the operating theatre. Quality and Safety in Health Care, 2009 18(2): p. 104–108. 10.1136/qshc.2007.024760 19342523

[16] Boaden R, Harevy G & Moxham C. *Quality Improvement*: *Theory and Practice in Healthcare*. 2008, Coventry, England: NHS Institute for Innovation and Improvement.

[17] Radnor Z.J., Holweg M., and Waring J., Lean in healthcare: the unfilled promise? Social Science & Medicine, 2012 74(3): p. 364–371. 21414703 10.1016/j.socscimed.2011.02.011

[18] Waring J.J. and Bishop S, Lean healthcare: rhetoric, ritual and resistance. Social science & medicine, 2010 71(7): p. 1332–1340. 20702013 10.1016/j.socscimed.2010.06.028

[19] Boaden R., *The contribution of quality management to patient safety*. Patient Safety: Research Into Practice, 2005: p. 41.

[20] Mazzocato P, Holden RJ, Brommels M et al, How does lean work in emergency care? A case study of a lean-inspired intervention at the Astrid Lindgren Children's hospital, Stockholm, Sweden>. BMC Health Serv Res, 2012 12: p. 28 10.1186/1472-6963-12-28 22296919 PMC3298466

[21] Burgess N. and Radnor Z., Evaluating Lean in healthcare. Int J Health Care Qual Assur, 2013 26(3): p. 220–35. 23729126 10.1108/09526861311311418

[22] Smith ML, Wilkerson T, Grzybicki DM et al, The effect of a lean quality improvement implementation program on surgical pathology specimen accessioning and gross preparation error frequency. American journal of clinical pathology, 2012 138(3): p. 367–373. 10.1309/AJCP3YXID2UHZPHT 22912352

[23] Kreckler S, Morgan RD, Cathpole K et al, Effective prevention of thromboembolic complications in emergency surgery patients using a quality improvement approach. BMJ quality & safety, 2013 22(11): p. 916–922. 10.1136/bmjqs-2013-001855 23708440

